# Acceptability and Feasibility of Using Hair Samples for Chronic Stress Measurement Among Transgender Women in Brazil

**DOI:** 10.1002/brb3.70156

**Published:** 2024-11-17

**Authors:** Sophia Zamudio‐Haas, Paula Galdino Cardin de Carvalho, Alexander Marr, A. Rain Mocelle, Antonio Moscatelli, Katia Cristina Bassichetto, Gustavo Santa Roza Saggese, Patric V. Prado, Roy Gerona, Sheri A. Lippman, Maria Amélia Veras, Jae M. Sevelius

**Affiliations:** ^1^ Department of Medicine University of California, San Francisco—UCSF San Francisco California USA; ^2^ Faculdade de Ciências Médicas da Santa Casa de São Paulo Sao Paulo Brazil

**Keywords:** Brazil, LGBT health, stress measurement, transgender women

## Abstract

**Introduction:**

The ability to objectively measure chronic stress has important implications for research, prevention, and treatment. Cortisol is currently the most used biological marker in the investigation of stress and can be measured via blood, saliva, and urine; however, these methods have disadvantages. The measurement of cortisol in hair is a more recently developed method that quantifies the cumulative production of cortisol over longer periods of time. Given the potential benefits of hair as a chronic stress biometric, research with this novel method is burgeoning, yet rarely involves transgender (“trans”) populations, despite high levels of reported stress among trans people due to experiences of stigma and discrimination. Since hair is a key part of gender presentation, trans people might be less likely than cisgender people to donate hair for research. To explore the feasibility and acceptability of hair collection for use as a stress biometric with trans women, we nested a study into an ongoing clinical trial in São Paulo, Brazil, “*Manas por Manas*” (Sisters for Sisters). Here, we describe the hair biometric substudy protocol, as well as the feasibility and acceptability of collecting hair in the study cohort.

**Methods:**

We randomly selected a subsample (*n* = 180) from the *Manas por Manas* cohort (*n* = 392), all of whom are trans women, age 18 or older. We messaged participants via phone, WhatsApp, or social media for at least three attempted contacts. Study visits included four components: (1) video introduction, including a demonstration of hair sampling; (2) informed consent; (3) a brief survey with the validated Short Stress Overload Scale (translated to Portuguese) and questions on hair care that could moderate stress hormone results; and (4) hair sample collection. Hair samples were collected and stored using validated protocols. Participants were reimbursed for travel costs.

**Results:**

Between April and December 2022, we messaged with 143 individuals out of the 180 sampled (79%) and invited them to participate in the study. Of those invited to participate, we scheduled study visits with 102 people (71.3% of those invited to participate), of whom 100 attended their study visits and completed all activities. Two people did not attend their study visits and stopped communication. Of those who were invited to participate and declined a study visit, four individuals declined due to the hair sample collection procedures (2.8% of those invited to participate). Other reasons for declining to participate included having moved (*n* = 7), lack of time (*n* = 11), not interested in research participation (*n* = 8), or unknown/stopped responding to messages (*n* = 11). Most participants reported that they chemically treated their hair to bleach, color, or straighten it, which could impact laboratory assays.

**Conclusion:**

We found hair sampling for stress measurement to be feasible and acceptable to our participants. We successfully completed all study activities for our desired sample size, and most recruited individuals volunteered to participate. Reasons provided for declining study participation reflected general barriers to research participation, with only four people declining due to hair sample collection procedures.

## Graphical abstract

Hair has promising benefits as a chronic stress biometric, and research with this novel method is burgeoning, yet rarely involves transgender (“trans”) people, despite high levels of reported stress from experiences of stigma and discrimination. Since hair is a key part of gender presentation, trans women might be more hesitant than cisgender women to donate hair for research. To explore the feasibility and acceptability of hair collection with trans women, we nested a study into an ongoing clinical trial in São Paulo, Brazil, “*Manas por Manas*” (Sisters for Sisters). We randomly selected a subsample (*n* = 180) from the *Manas* cohort (*n* = 392. We invited 143 trans women to participate, of whom 100 attended their study visits and completed all activities. Of those who declined participation, only four individuals said it was because of the hair sample collection. Given these results, we found hair sampling for stress measurement to be feasible and acceptable.

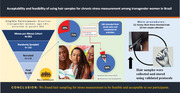

## 1 Introduction

Prolonged, persistent, or recurring stress has a range of negative impacts on physical and mental health (Adam et al. 2017; Marin et al. 2011; Yaribeygi et al. 2017), yet objectively measuring chronic stress poses methodological challenges. Chronic stress has a bi‐directional relationship with emotional health (Joseph and Golden 2017; Marin et al. 2011) and cognition (Marin et al. 2011; Sterlemann et al. 2010), such that chronic stress can result from emotional health challenges following early life adversities (Khoury et al. 2019), workplace burnout, and experiences of stigma and discrimination (Staufenbiel et al. 2013), while it can also contribute to the development and exacerbation of mental illnesses. For physical health, chronic stress contributes to higher risk of, and worse prognosis for, cardiovascular disease (Kivimäki and Steptoe 2018; Kwok et al. 2020; Osborne et al. 2020; Steptoe and Kivimäki 2012), obesity (Adam et al. 2017; Hewagalamulage et al. 2016; Roy et al. 2021), Type 2 diabetes (Adam et al. 2017; Joseph and Golden 2017; Kwok et al. 2020), chronic obstructive pulmonary disorder (Du et al. 2014), skin diseases (Pondeljak and Lugović‐Mihić 2020), irritable bowel syndrome (Pellissier and Bonaz 2017; Qin et al. 2014), and other health outcomes. Chronic stress can decrease memory capabilities and cognition (Schwabe et al. 2022; Sousa et al. 2021).

The ability to measure chronic stress has important implications for research, prevention, and treatment. For research, biomarkers could help researchers objectively and reliably investigate the effects of stress on health and test the efficacy of interventions to mitigate or reduce stress. Early detection of chronic elevated stress can raise awareness of stress before health impacts appear (Iob and Steptoe 2019), helping to prevent the long‐term health consequences of chronic stress. The ability to measure and monitor changes in chronic stress over time can complement and validate self‐reported experiences and support individualized treatment efforts (Wosu et al. 2013). While a strong and validated evidence base provides self‐reported psychological measures for stress related to work environment (Griep et al. 2009; Stanhope 2017), relationship stress (Bowman 1990; Rahim 1983; Straus and Mickey 2012), and stigma and discrimination (Contrada et al. 2001; Page‐Gould, Mendoza‐Denton, and Mendes 2014); cumulative life stress (Slavich and Shields 2018) remains challenging to measure, due to challenges accounting for independent interrelationships between risk factors and multiplicative effects from interactions (Evans, Li, and Whipple 2013). Measurement research around intersectional stigma suggests that people experiencing multiple forms of chronic stigma might underreport their experiences as compared to those experiencing stigma due to one characteristic, further complicating self‐report measurements around stress for transgender women of color (Balsam et al. 2011; Guan et al. 2021; Wesson et al. 2021).

Cortisol is currently the most used biological marker in the investigation of chronic stress (Russell et al. 2012) and can be measured via blood, saliva, and urine; however, these methods have disadvantages (Greff et al. 2019). Blood, saliva, and urine are limited to measures of acute cortisol secretion over a short period, leading to a need to perform multiple collections over the period of study to minimize the circadian effect (i.e., acute variations in cortisol production throughout the day) (Greff et al. 2019; Krieger et al. 1971). In addition, blood, urine, and saliva samples require refrigeration and cold chain transportation (Lee, Kim, and Choi 2015). Blood collection is an invasive procedure, which requires additional biohazard precautions for storage and transportation (Lee et al. 2015). Collection methods for all three biometrics can induce stress and increase the secretion of cortisol during collection, thereby changing the results (Greff et al. 2019; Lee et al. 2015; Russell et al. 2012).

The measurement of cortisol in hair is a more recently developed method that quantifies the cumulative production of cortisol over longer periods of time (Bévalot et al. 2000; Raul et al. 2004; Russell et al. 2012; Stalder et al. 2017). This method minimizes the circadian effect and bias from collection procedures. Hair collection is non‐invasive, requiring only a small sample of as few as 30 strands (Greff et al. 2019). Storage and transport of hair has no special requirements (Greff et al. 2019); hair is stored at room temperature, and cortisol present in hair will remain stable for years (Gow et al. 2010).

Given the potential benefits of hair as a chronic stress biometric, research with this novel method is burgeoning, yet rarely involves transgender (“trans”) populations (Rodríguez Madera et al. 2017), despite high levels of reported stress among trans people (Delozier et al. 2020; DuBois et al. 2017; Zoccola et al. 2017). Hair holds an important role in gender presentation and expression (Bared and Epstein 2023; Gao, Maurer, and Mirmirani 2018; Rossiter 2016), and trans women might have unique concerns or hesitancies about providing a hair sample. In formative research with trans women in Puerto Rico, trust proved essential to participants’ decision to donate hair for analysis (Rodríguez Madera et al. 2017). Research using hair as a biometric for PrEP adherence found that trans women were willing to participate and donate hair, yet also noted that 25% of all people who declined were “worried about hairstyle disruption” (Gandhi et al. 2017).

To explore the feasibility and acceptability of hair collection for use as a stress biometric with trans women, we nested a study into an ongoing clinical trial with trans women in São Paulo, Brazil, “*Manas por Manas* (Sisters for Sisters)” (NCT04114955 2023). *Manas por Manas* (hereafter “*Manas*”) is a randomized waitlist‐controlled trial to assess the impact of a peer support intervention designed to address intersectional stigma and increase HIV prevention behaviors among trans women. We describe the hair biometric substudy protocol as well as the feasibility and acceptability of collecting hair.

## 2 Methods

### 2.1 Study Setting

We conducted data collection at one of the *Manas* (NCT04114955 2023) study sites located in downtown São Paulo and accessible via subway and bus lines. Study staff created a welcoming and comfortable space for participant visits, with couches, light refreshments, and images celebrating trans people and promoting LGBT health on the walls.

### 2.2 Sample Selection and Recruitment

We performed multiple rounds of simple random sampling (total *n* = 180) from enrolled *Manas* participants (*n* = 392), who are trans women, age 18 or older, until we achieved our target enrollment. The participant cohort enrolled in *Manas* from which we sampled participants for the substudy had all completed their baseline surveys and were drawn evenly from the comparison and intervention groups. The coordinator contacted selected participants via phone, WhatsApp, or social media for at least three attempted contacts. However, unless we received a message from the participant indicating they were not interested, the coordinator would pause for a few weeks and then reach out again. Once we established communication, the coordinator scheduled a study visit at the participant's convenience.

### 2.3 Study Components

Study visits for the hair stress analysis substudy were scheduled independently of activities for the *Manas* parent study and included four components and lasted about an hour. (1) Introduction—participants were shown a video describing the study and an example of a hair sample to provide a visual reference for the size of the lock that would be collected. (2) Informed consent—the coordinator administered informed consent and answered any questions. (3) Generalized stress survey—the coordinator interviewed participants using a closed‐ended, validated Short Stress Overload Scale (Amirkhan 2018). The survey included additional questions about hair care to capture elements that could influence the quality of the hair sample or cortisol measurement, such as frequency of washing (Hamel et al. 2011), dying or straightening (Hoffman et al. 2014; Kristensen et al. 2017), and use of cortisol topical cream. (4) Hair sample collection—a lock of hair close to the scalp was cut following validated protocols for collection and storage (Gandhi et al. 2017; Saberi et al. 2018).

### 2.4 Measures

#### 2.4.1 Acceptability

We measured acceptability by documenting the willingness of recruited participants to complete all study activities. If recruited potential participants declined to participate, we asked why they declined and noted this in our study tracking log. Once participants provided informed consent, we further measured acceptability by calculating the proportion of study participants completing hair collection, again noting if participants declined and their reason. We also asked each participant how it went after the hair collection (“How are you feeling? How did it go?”) to gather systematic feedback on their study experience and recorded notes on a spreadsheet. Study visit notes were analyzed via review of the spreadsheet, with attention to negative feedback on hair collection procedures, discussion of stress and stress measurement, and suggestions on how to improve study procedures. Notes around recruitment were analyzed separately, with attention to tabulating the number of people who declined participation and reasons why, people who stopped communicating with the study team, and people who never responded to our initial recruitment messages.

#### 2.4.2 Feasibility

We measured feasibility by documenting our ability to recruit and enroll participants according to the study timeline (about six months) and within the allotted effort of the study coordinator (12 h per week). We further measured feasibility by documenting ease or difficulty of procedures for collection of hair and survey data (i.e., training, materials, and storage) on a study participant visit spreadsheet. We aimed for 90% of hair samples to be suitable for analysis (i.e., properly labeled and stored) and carefully inspected each specimen before analysis. We also measured feasibility by documenting the time it took for each visit and the time it took to train study staff.

### 2.5 Statistical Analysis

We conducted appropriate tests of correlation to assess for differences between *Manas* participants enrolled in the stress substudy (*n* = 100) and the rest of the *Manas* cohort (*n* = 292) for several key indicators, such as demographics, housing stability, alcohol and recreational drug use, the Kessler 10‐item psychological distress scale (Treharne et al. 2020), and suicidal ideation.

### 2.6 Ethics

Study protocol was approved by the University of California, San Francisco Institutional Review Board in the United States, and the National Ethics Committee in Brazil. Participants were reimbursed for travel costs (R$50 or about US$9.70). All participants provided informed consent to participate. Study staff completed training in research with human subjects.

## 3 Results

We found no significant differences in key demographics, behavioral, and stress indicators between the substudy group and those not included from the *Manas* cohort (Table 1).

**TABLE 1:** Comparison of substudy participants and remaining *Manas* study cohort.

Baseline characteristics	Stress study (*N* = 100)	Remaining *Manas* study cohort (*N* = 292)	*p* value
	*N* (%)	*N* (%)	
Age (mean, 95% CI)	33.9 (32.0–35.8)	33.2 (32.1–34.4)	0.55
18–24 years	17 (17.0)	58 (19.9)	
25–35 years	41 (41.0)	130 (44.5)	
> 35 years	42 (42.0)	104 (35.6)	
Race/ethnicity			
Black	32 (32.0)	75 (25.7)	0.15
White	29 (29.0)	70 (24.0)	
Mixed/other race	39 (39.0)	147 (50.3)	
Birth place			
São Paulo	40 (40.0)	127 (43.5)	0.54
Other	60 (60.0)	165 (56.5)	
Stable housing			
Yes	77 (77.0)	211 (72.3)	0.35
No	23 (23.0)	81 (27.7)	
Psychological distress			
Severe distress	40 (40.0)	125 (42.8)	0.62
None to moderate distress	60 (60.0)	167 (57.2)	
PTSD			
Yes	65 (65.0)	173 (59.2)	0.31
No	35 (35.0)	119 (40.8)	
Suicidal ideation ever			
Yes	59 (59.0)	195 (66.8)	0.16
No	41 (41.0)	97 (33.2)	
Audit‐C designation			
Hazardous drinker	54 (54.0)	154 (52.7)	0.70
Not hazardous	45 (45.0)	138 (47.3)	
Illegal drug use in the previous 12 months			
Yes	53 (53.0)	152 (52.1)	0.87
No	47 (47.0)	140 (47.9)	

## 4 Acceptability

### 4.1 Recruitment and Enrollment

We conducted recruitment and enrollment on an ongoing basis between April and December 2022, until we successfully completed all study visits (Table 2). We systematically messaged people on our list of randomly selected *Manas* participants, recruiting and setting up study visits with those who responded to our messages and were interested in learning more about the study. Once we completed our target number of study visits (*n* = 100), we stopped contacting participants. In total, we messaged 169 individuals (93%) out of the 180 randomly selected *Manas* participants. We did not attempt to recruit the remaining 7%, as we had already achieved desired enrollment. Of the 169 people to whom we sent an introductory message, 143 people (84%) responded and were invited to participate in the study. We scheduled study visit appointments with 102 people, of whom two did not present for their appointment. Our total planned sample size of 100 people attended their scheduled visits, enrolled in the study, and completed all study components.

**TABLE 2:** Recruitment and enrollment.

Recruitment activity	*N*	%
Random sample drawn from *Manas* cohort	180	
Attempted to contact	169	93% of those sampled
Unable to reach (no response)	26	15% of attempted to contact
Invited to participate	143	85% of attempted to contact
Declined participation	41	29% of those invited
Reason for declining participation		
Recently shaved head	1	2.4% of those declined
Due to hair collection procedures	3	7.3% of those declined
Moved outside of São Paulo	7	17.1% of those declined
Lack of time	11	26.8% of those declined
Reason not given	19	46.3% of those declined

Of the 41 people who declined a study visit, only four people cited reasons related to the hair sampling procedures, and, of those, one declined due to a shaved head hairstyle that made sample collection impossible. Other reasons for nonparticipation included having moved outside of São Paulo (*n* = 7), no time to attend (*n* = 11), and disinterest in participation (*n* = 8). Eleven people did not provide a reason for declining a study visit; however, they stopped responding to messages from the study coordinator, suggesting a tacit declination.

### 4.2 Completion of Study Timeline

Study activities were successfully completed according to the study timeline, indicating that this method of biomarker collection can be feasibly conducted with reasonable effort. We had planned for 6 months for recruitment and study visits, with one research coordinator leading recruitment and data collection activities on a part‐time basis (25% effort). This amount of time was sufficient to conduct recruitment and study activities, with each study visit lasting about an hour.

The recruitment process worked effectively, demonstrated by the similarities in the substudy population who completed activities and the rest of the Manas cohort (Table 1). The randomization appears successful, regardless of the nonresponses, declined study visits, and two people who did not attend their visits. A comparison on key demographic, behavioral, and stress indicators revealed no significant differences between those who donated hair and others enrolled in the parent study.

### 4.3 Ease of Study Procedures

Study procedures were easy to learn based on feedback from study staff. We conducted an online training, which was about an hour, on the overall study procedures (recruitment, enrollment, and study components). We additionally conducted an in‐person training on hair sample collection and tested the procedures with a volunteer. The hair sample was easy to collect, label, and store, requiring no special conditions for storage (i.e., no refrigeration needed or biohazard precautions). Materials needed to conduct the hair sample collection were minimal and inexpensive, including small scissors to cut the hair sample and aluminum foil, labels, and plastic zipper bags to store the samples. All hair samples received at the laboratory were correctly packaged and labeled. After a careful review of each specimen, the laboratory successfully analyzed the cortisol and cortisone levels in all hair samples.

#### 4.3.1 Limitations

Our substudy sampled and recruited participants who were enrolled in an on‐going clinical trial. Thus, our findings are biased towards feasibility and acceptability, as we are drawing from a group of trans women who had already demonstrated willingness to participate in a research study. It is possible that we would have observed lower acceptability or have needed to sample more people to recruit to reach out target enrollment, if we were recruiting from a population who were not currently enrolled in a research study.

## 5 Conclusions

We found hair sampling for stress measurement to be feasible to implement and acceptable to study participants: a cohort of Brazilian trans women enrolled in an HIV prevention clinical trial. Of the participants successfully contacted and invited to participate, 70% completed study visits. All participants who presented for their study visits completed the survey and hair collection, showing a high degree of acceptability of procedures. Only four people of the 143 who we messaged with about the study declined participation due to the hair collection methods.

In other studies collecting hair as a biometric, participation has ranged from 58% (Gandhi et al. 2017) to 95% (Koss et al. 2018), depending on the study protocol (Barbosa et al. 2013; Gerona et al. 2016; Musana et al. 2022; Saberi et al. 2018). Notably, with one exception (Gandhi et al. 2017), these previous studies did not include transgender participants. Given the importance of hair in gender presentation, we developed procedures aimed at addressing concerns that we anticipated might reduce participation levels. To increase the acceptability of procedures, we provided ample training around hair collection so that the study coordinator was well prepared to address common concerns around hair collection during recruitment and consent, such as the time that it takes to collect the sample, the amount of hair collected, and the ability to collect a sample from people with short hair or hair extensions (Saberi et al. 2018). We also recruited from a group of people who already had enrolled in a study through NUDHES, which could in part explain our high acceptability. NUDHES has a history of conducting community‐engaged research (Amarante et al. 2023; Lippman et al. 2022) with transgender people in São Paulo, which could increase trust with participants, a factor previously identified as important for facilitating hair collection with transgender women (Rodríguez Madera et al. 2017).

Our results suggest that hair sample collection with transgender women is highly feasible. We successfully recruited, enrolled, and completed data collection with 100 participants as planned, within the target timeframe. As other studies have shown, hair collection is painless and quick, with samples easily stored and transported (Gandhi et al. 2017; Gerona et al. 2016; Saberi et al. 2018). Study staff quickly mastered the procedures for cutting and storing hair samples. All samples were properly labeled and successfully analyzed.

This feasibility and acceptability study is a first step towards examining the utility of hair as a chronic stress biometric for mental health research with trans women, who navigate high amounts of daily stress in their lives (Bockting et al. 2013; Gamarel et al. 2016; Wilson et al. 2015), but who also could be less amenable to hair collection. Given the high levels of stress that trans women experience over the life course (Gamarel et al. 2016; Wilson et al. 2015), there is an urgent need to develop and tailor mental health interventions to build resilience, increase coping, and promote wellbeing for this important and unique population, as well as a need for metrics to document intervention impacts. These high levels of lifetime stress might complicate the viability of biomarkers that have been studied primarily with cis‐gender populations, such as allostatic load (Juster et al. 2016; Sterling 1988), increasing the importance of stress biomarker research with trans communities, who are often left out of mental health research study populations.

Our findings demonstrate the feasibility of hair collection for stress measurement with a unique study population of Brazilian transgender women. Conversations during study visits suggested that participants are interested in talking about stress in their lives and that it is a priority health topic. Many shared that transphobia exacerbated other sources of stress in their lives around relationships, employment, and housing. Deepening understanding of sources of stress and effective strategies to mitigate and measure stress are important areas for further research with trans women in Brazil.

## Author Contributions

**Sophia Zamudio‐Haas**: conceptualization, investigation, funding acquisition, writing–original draft, methodology, writing–review and editing, supervision, visualization, formal analysis, project administration, data curation, resources. **Paula Galdino Cardin de Carvalho**: investigation, writing–original draft, writing–review and editing, data curation, supervision, project administration, resources, formal analysis, methodology. **Alexander Marr**: methodology, visualization, formal analysis, data curation, validation, writing–review and editing; writing–original draft. **A. Rain Mocelle**: methodology, validation, writing–review and editing, formal analysis, supervision, data curation. **Antonio Moscatelli**: formal analysis, writing–original draft, writing–review and editing, methodology. **Katia Bassichetto**: formal analysis, data curation, project administration, writing–review and editing, methodology. **Gustavo Saggese**: writing–review and editing, funding acquisition, methodology, project administration, supervision, resources, investigation. **Patric Prado**: validation, methodology, visualization, writing–review and editing, formal analysis, data curation, conceptualization, funding acquisition. **Roy Gerona**: methodology, investigation, conceptualization, funding acquisition, writing–review and editing, supervision, data curation. **Sheri Lippman**: conceptualization, investigation, funding acquisition, writing–review and editing, supervision. **Maria Veras**: conceptualization, investigation, funding acquisition, writing–review and editing, methodology, supervision. **Jae M. Sevelius**: conceptualization, investigation, funding acquisition, writing–review and editing, supervision.

### Peer Review

The peer review history for this article is available at https://publons.com/publon/10.1002/brb3.70156.

## Data Availability

Data will be made available upon reasonable written request.
